# Quantitative assessment of microbicide-induced injury in the ovine vaginal epithelium using confocal microendoscopy

**DOI:** 10.1186/1471-2334-12-48

**Published:** 2012-02-29

**Authors:** Gracie Vargas, Igor Patrikeev, Jingna Wei, Brent Bell, Kathleen Vincent, Nigel Bourne, Massoud Motamedi

**Affiliations:** 1 Center for Biomedical Engineering, The University of Texas Medical Branch, Galveston, TX, USA; 2 Department of Neuroscience and Cell Biology, The University of Texas Medical Branch, Galveston, TX, USA; 3 Department of Pediatrics, The University of Texas Medical Branch, Galveston, TX, USA; 4 Department of Obstetrics and Gynecology, The University of Texas Medical Branch, Galveston, TX, USA; 5 Department of Opthalmology, The University of Texas Medical Branch, Galveston, TX, USA

**Keywords:** Benzalkonium chloride, Confocal endomicroscopy, Epithelial disruption, Imaging, Microbicides, Nonoxynol-9

## Abstract

**Background:**

The development of safe topical microbicides that can preserve the integrity of cervicovaginal tract epithelial barrier is of great interest as this may minimize the potential for increased susceptibility to STI infections. High resolution imaging to assess epithelial integrity in a noninvasive manner could be a valuable tool for preclinical testing of candidate topical agents.

**Methods:**

A quantitative approach using confocal fluorescence microendoscopy (CFM) for assessment of microbicide-induced injury to the vaginal epithelium was developed. Sheep were treated intravaginally with one of five agents in solution (PBS; 0.02% benzalkonium chloride (BZK); 0.2% BZK) or gel formulation (hydroxyethyl cellulose (HEC); Gynol II nonoxynol-9 gel (N-9)). After 24 hours the vaginal tract was removed, labeled with propidium iodide (PI), imaged, then fixed for histology. An automated image scoring algorithm was developed for quantitative assessment of injury and applied to the data set. Image-based findings were validated with histological visual gradings that describe degree of injury and measurement of epithelial thickness.

**Results:**

Distinct differences in PI staining were detected following BZK and N-9 treatment. Images from controls had uniformly distributed nuclei with defined borders, while those after BZK or N-9 showed heavily stained and disrupted nuclei, which increased in proportion to injury detected on histology. The confocal scoring system revealed statistically significant scores for each agent versus PBS controls with the exception of HEC and were consistent with histology scores of injury.

**Conclusions:**

Confocal microendoscopy provides a sensitive, objective, and quantitative approach for non-invasive assessment of vaginal epithelial integrity and could serve as a tool for real-time safety evaluation of emerging intravaginal topical agents.

## Background

Effective preclinical microbicide safety testing is of great importance to insure that only the most promising candidates are advanced to clinical trials. This is particularly important in light of the fact that several clinical trials of emerging microbicides have been terminated due to safety concerns [1-3]. Several early microbicide candidates were shown to increase susceptibility to infection *in vivo *[4,5] and were associated with surface epithelial disruption and inflammation [4-6]. Additionally, undetected changes in the epithelial integrity below the detection threshold of traditional safety assessment techniques such as colposcopy may yet increase susceptibility to infection [2]. Tools which aid in noninvasive visualization of the vaginal mucosa and effects of agents at the microscopic level in real time could greatly benefit the microbicide field. Such capabilities could facilitate the design of safe and effective topical agents.

A number of methods are currently used for assessing microbicide effects on the cervicovaginal tract. Colposcopy is easily implemented and allows repeated evaluation. Disadvantages are it only allows visual assessment of the tissue surface albeit with magnification, colposcopy training requirements are intensive, and interpretation is subjective and only modestly quantitative. Other currently used methods to detect epithelial damage and inflammation do not provide immediate feedback regarding the effect of an agent. Histopathology requires biopsy and processing of tissue and results in days to weeks of delay. Thus, it is both cumbersome and raises safety issues when used clinically in high-risk populations. Cytokine mapping following vaginal lavage requires offline testing, but has the advantage of being compatible with other methods and can be repeated in longitudinal studies.

Recently, noninvasive imaging has been investigated for the *in vivo *near microscopic visualization of the vaginal epithelium in small and large animal models. Optical coherence tomography (OCT), an imaging tool applied to the mouse and ovine vaginal epithelium, provides quantitative measurement of epithelial thickness changes at a resolution of approximately 10-15 μm and has been used to detect microbicide-induced changes in epithelial thickness and morphology following topical application of BZK to the ovine vaginal tract [7]. Although OCT allows for imaging of the full vaginal mucosa and submucosa, the resolution does not allow for evaluation of subcellular changes or cell viability assessment.

CFM is a high resolution noninvasive imaging method commercially available as an endoscopic system [8]. It has been used in a number of clinical cancer trials primarily in the gastrointestinal tract and lung in studies using systemic or topically applied fluorophores to reveal morphological or cytological abnormalities on the epithelial surface during real-time endoscopy [8-10]. In the colorectum, CFM detected architectural differences between the normal crypt structure and neoplasia [11,12]. In the esophagus it is being investigated as an adjunct tool in the management of precancerous lesions [13,14]. Although CFM has not been used in clinical studies in the cervicovaginal tract it has been used in preclinical studies in sheep to examine cellular morphology changes on the cervical surface following progesterone and estradiol hormonal treatment [15]. In the current study, we investigate the ability of a fixed-plane surface imaging CFM to visualize and quantify epithelial injury in the ovine cervicovaginal tract following treatment with solutions and gels commonly studied in microbicide research.

## Methods

### Animal model and in vivo agent application

Studies were approved by the Institutional Animal Care and Use Committee at the University of Texas Medical Branch and conformed to the Guide for the Care and Use of Laboratory Animals. Virginal Rambouillet female sheep (n = 17 weighing 25-35 kg) were sedated with 10 mg/kg ketamine IM followed by IV administration of 10 mg/kg ketamine and 0.1-0.2 mg/kg Diazepam to relax the vaginal sphincter. The animals were placed dorsal supine on a V-tilt table and intubated for delivery of isoflurane throughout microbicide treatment and imaging procedures.

The cervicovaginal tract was examined using a pediatric Graves speculum and the animals were administered 8 cc of one of the following test agents: PBS (n = 3), Gynol II, an over the counter spermicidal gel containing 2% N-9 (n = 4), HEC regarded as a universal placebo gel (n = 4) [16], 0.02% BZK solution (n = 3), or 0.2% BZK solution (n = 3). Test agents were coded to mask investigators to treatments, then were delivered intravaginally using a ball-tip catheter. After twenty-four hours, the animal was sacrificed and the vaginal tract immediately removed for imaging.

### Imaging

The CFM used in these studies provided excitation at 488 nm through a fiber bundle with fluorescence emission collected in the spectral band of 505-700 nm (Cellvizio Lab, Mauna Kea, France). The system lateral resolution is 3.3 μm and field of view is 600 × 600 μm. The 1.5 mm diameter endoscopic probe requires contact to obtain images of the surface in a plane parallel to the surface (*en face*) and has an axial resolution of 15 μm. A single optically sectioned plane was obtained at the surface. Images were acquired at a rate of 12 frames per second. Individual frames were extracted in TIFF format.

Each freshly excised vaginal tract was opened longitudinally along the anterior wall and laid flat with the mucosal surface exposed. The surface was rinsed with PBS and 20 μM propidium iodide (PI) applied evenly to the surface using a bulb pipet. PI is a fluorescent dye that binds to DNA and is not permeant to healthy cells. Thus damaged or dead cells were identified by fluorescence from the cell nuclei. After 5 minutes the PI solution was rinsed off using PBS. The CFM probe was placed directly on the vaginal surface and an image series collected site-by-site in a predetermined 5 × 5 matrix on the proximal vagina surface above the sphincter with a 1 cm separation between image sites. Following imaging, the entire tract was fixed in 10% formalin for 24 hours and then the imaged sites which had been marked were biopsied (2 mm), paraffin embedded, sectioned, and stained with hemotoxylin and eosin.

### Image analysis

An algorithm was developed to quantify the effects of topical agents on epithelial surface integrity based on the PI staining pattern. The algorithm incorporated feature size information through calculation of the mean-to-median size of all individual objects within an image. The basis for such an approach is that in the native epithelium, the nuclear staining pattern is expected to consist of intact nuclei of uniform size across the vaginal surface (these nuclei are considered the objects in the algorithm). In the case where there is no variation in size of objects, the calculated median size and mean size of objects (nuclei) are equal, or very closely match if there is a small variation in size. However, if the image is comprised of features which vary significantly in size, as might be expected if nuclear material aggregates following lysis, the median size of objects will increase relative to the mean size.

All analyses were performed with Matlab software version R2006a (Mathworks, Natick, MA) using standard operations of the image processing toolbox. A representative image was selected for each of the 25 vaginal sites per animal. Each raw image consisted of a range of grayscale pixel values with those belonging to the object bright in comparison to the surrounding dark pixels. Images were thresholded using a predetermined global threshold based on unstained control samples (in this process pixels with a value greater than the threshold value were assigned as 'object' pixels while those with values smaller than the threshold value as dark or subthreshold pixels). Binary images were created with pixels assigned a value of 0 or 1 to identify sub-threshold and above threshold (object) pixels, respectively. A Matlab labeling operation (bwlabel) was used to identify all individual objects within the image. This operation determines all clusters of interconnected pixels with a value of 1 and assigns each cluster (each object) a number. Using region statistics, the size (area) of each object was then determined. The median size of objects within the image and the mean size was then determined and a mean-to-median ratio calculated for the image. This was performed for all 25 image sites per animal, PBS treated controls were used to determine a typical value for mean-to-median ratio and a threshold value was set above this to differentiate 'normal' from 'abnormal'. The average mean-to-median value in PBS controls was found to be 1.5 and 90% of images had a mean-to-median ratio < 2.0. The threshold value differentiating normal from abnormal value was set to 2.0. The number of images in each animal having a mean-to-median ratio > 2.0 was then enumerated and an average determined per treatment group.

### Histological assessment

Photographs of H&E stained sections from biopsies of the 25 sampled regions were obtained using a color CCD mounted on an IX71 Olympus inverted microscope. Photographs were randomized and graded by a trained grader familiar with the assessment of BZK-induced damage in sheep vaginal epithelium but masked to the treatments in this study [7]. H&E sections with fully intact epithelium having a cornified stratified squamous epithelium of full thickness were given a score of 0. Samples exhibiting partial denuding, such as a visually thinned epithelium and evidence of inflammation determined by the presence of leukocytes, were given a score of 1. Samples with fully denuded epithelium were given a score of 2. A score was given to each of the 25 biopsy sites and used to determine the average score for each animal. These individual animal scores were then used to generate mean scores for each treatment group.

Epithelial thickness was measured at three predetermined locations on photographs of an H&E section from each imaged site. A single average per animal and per treatment group was then determined.

### Statistical analysis

Statistical analysis of confocal data for comparison between treatment groups was by single factor ANOVA followed by Tukey's *post hoc *test. A value of *p *< 0.05 was considered statistically significant. Single factor ANOVA with Tukey's *post hoc *test was also applied to epithelial thickness data. For statistical analysis of histology scores (an ordinal variable -0,1,2) the mixed model was used to test the treatment effects and perform multiple comparisons. Tukey adjustment was used for the *p*-values in the multiple comparisons.

## Results

**Figure 1:**
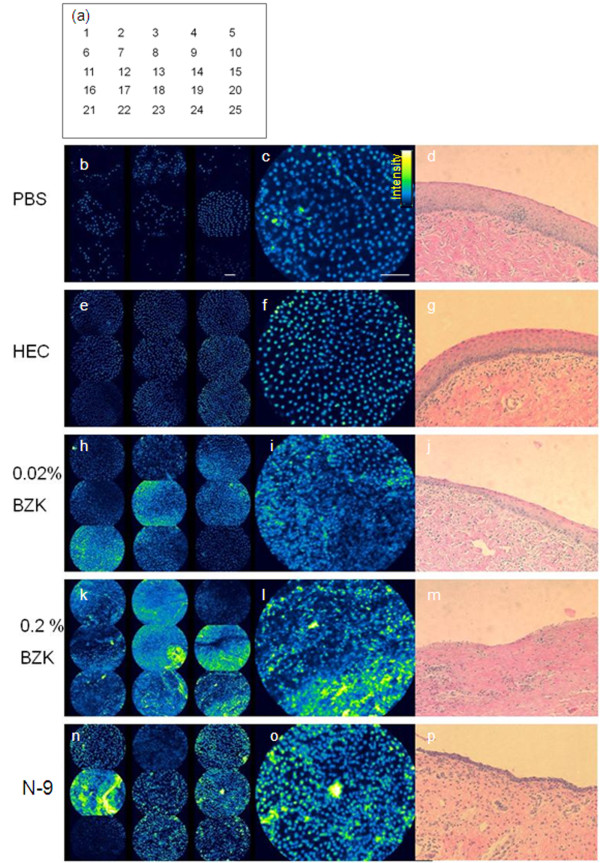
**Representative confocal microendoscopy and histology images**. For each treatment group, a 3 × 3 layout of 9 confocal images representing positions 1,3,5,11,13,15,21,23,25 of the sampled 5 × 5 image matrix from a single animal is shown (left column), followed by a single larger confocal image (center column), and corresponding H&E section (right column). (a) 5 × 5 matrix representing the positions of the 25 imaged sites per animal. (b-d) representative PBS case; (e-g) representative HEC treated case, (h-j) representative 0.02% BZK treated case; (k-m) representative 0.2% BZK treated case; (n-p) representative N-9 treated case. Confocal images are shown in pseudocolor for ease of visualization with the intensity represented by the shown color (black represents a zero intensity value, progressing to yellow for maximum intensity). Scale bar 100 μm.

**Figure 2:**
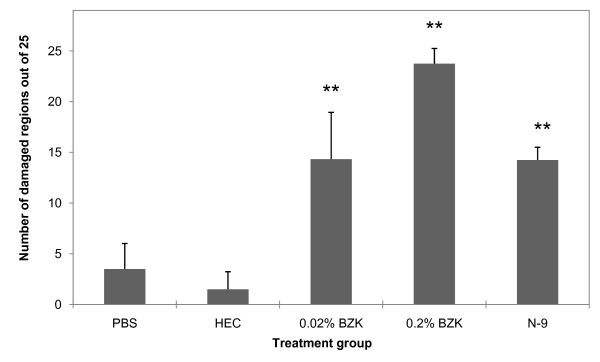
**Number of damaged regions based on mean-to-median ratio**. Images with a mean-to-median ratio were enumerated per animal and averaged within treatment groups. No statistical significance occurred between PBS and HEC cases. Statistically significant increase in the number of damaged regions occurred with 0.02% BZK, 0.2% BZK, and N-9. Results in the 0.02% BZK case were similar to N-9, and both were statistically different from those at 0.2% BZK. Statistical analysis for comparison between treatment groups was by single factor ANOVA followed by Tukey's *post hoc *test for significance. Statistical significance with *p *< 0.05 is represented with * and *p <*0.01 with **.

**Figure 3:**
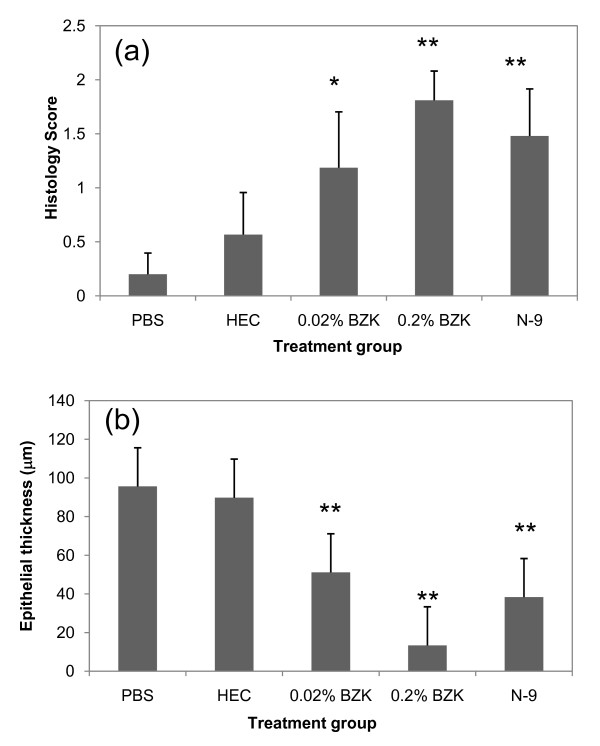
**Histology Scores and Epithelial thickness per treatment group**. (a) Results from visual scoring histological analysis of injury based on an ordinal value scoring system (0,1,2). Statistical analysis of ordinal variable (0,1,2) histology scores was by the mixed model to test the treatment effects and perform multiple comparisons. Tukey adjustment was used for the *p*-values in the multiple comparisons. (b) Epithelial thickness measured quantitatively on H&E histology single sections. Single factor ANOVA followed by Tukey's *post hoc *test was applied to test for statistical significance. Statistical significance with *p *< 0.05 is represented with * and *p *< 0.01 with **.

Representative CFM and histology images for each treatment are shown in Figure 1. The confocal images are shown in pseudocolor rather than grayscale for ease of visualization. The intensity is represented by a green-blue fire look-up table color scale (black represents zero intensity progressing to blue, green, yellow, white for maximum intensity as shown in the intensity scale of Figure 1). Each representative layout shows confocal images of 9 of 25 sampled regions (positions 1,3,5,11,13,15,21,23,25 of the 5 × 5 image matrix) from a single animal in the left column, a single larger confocal image in the center column, and a corresponding H&E image in the right column. Confocal images from PBS treated animals showed uniformly-sized nuclei distributed throughout the surface. Nuclei had well defined borders. Fluorescent staining in the PBS controls is attributed to the cornified surface layer from which cells are shed throughout the estrus cycle. Histology from PBS treated sheep confirmed the presence of a cornified stratified squamous epithelium (Figure 1). The PI staining pattern and histology following treatment with HEC placebo gel were similar to that in PBS controls (Figure 1e f and 1g). In contrast, treatment with BZK (0.02% or 0.2%) or N-9 resulted in a different staining pattern in confocal images and altered epithelium in histology as can be visualized in Figure 1 and shown quantitatively in Figures 2 and 3. Following treatment with 0.02% BZK there was an increase in the total area of staining, and although individual nuclei could still be identified, they were more closely spaced and merged in areas likely due to cell lysis (Figure 1h and 1i). Corresponding histology (Figure 1j) showed a thinned epithelium and inflammatory infiltrates. Treatment with 0.2% BZK produced increased staining with disruption of the regular nuclear spacing and presence of nuclear aggregate/debris in confocal micrographs (Figure 1k and 1l). Corresponding histology revealed extensive epithelial damage with complete denuding in most samples (Figure 1m). Results following N-9 treatment were somewhat similar to 0.02% BZK in that there was increased staining and some disruption to the regular distribution of nuclei across the sampled regions (Figure 1n and 1o). Much of the epithelium became denuded as well (Figure 1p).

Figure 2 shows the results from the mean-to-median ratio analysis per treatment group. In PBS animals, the average mean-to-median value was found to be 1.5. Results in HEC treated animals were comparable. However, N-9, 0.02% BZK, and 0.2% BZK, treatment all resulted in statistically significant changes. Corresponding histology scores and epithelial thicknesses are shown in the plots of Figure 3a and 3b. In both cases, statistically significant differences were also detected between PBS and N-9, 0.02% BZK, and 0.2% BZK, but not between PBS and HEC.

## Discussion

In evaluating safety of candidate microbicides, there is a need to identify indicators of epithelial damage which may signify increased susceptibility to cervicovaginal infection with STI pathogens. There is evidence to suggest that disruption of the epithelial barrier is linked to increased susceptibility [5]. However, susceptibility studies are not practical for initial agent testing in large animal models due to the high cost of studies. Thus, direct imaging that provides feedback regarding epithelial integrity/disruption could be valuable in preclinical testing of candidate topical agents. In the current study CFM provided real-time high resolution single-plane visualization of the vaginal surface epithelium and utilizing an automated analysis algorithm developed for this work, provided assessment of damage based on degree of positive staining by a cell impermeant nucleic acid dye. To our knowledge, this is the first study to investigate this imaging modality for this purpose.

Two agents were evaluated that have no known effect on epithelial structure (PBS and HEC) and two agents (BZK and N-9) that have been shown previously to induce cervicovaginal epithelial disruption in small and large animal models [5-7,17]. Because the use of 2% BZK has been shown to lead to immediate widespread epithelial exfoliation and complete denuding of the epithelium [5,7], we chose to use lower dilutions of BZK at 0.02% and 0.2% for this study. For the N-9 gel we chose an over-the-counter formulation shown to produce epithelial disruption [1,2]. CFM revealed differences in the PI staining patterns of vaginal tissue treated with N-9 and BZK compared to PBS. While there was a regular baseline staining pattern of nuclei across the surface in PBS controls (attributed to cornified cells about to be shed from the surface and permeant to PI), there was a clear increase in nuclear density with small degrees of damage (e.g. 0.02% BZK; Figure 1h and 1i) and evidence of exfoliated material that formed large aggregated nuclear material in confocal micrographs (e.g. 0.2% BZK; Figure 1k and 1l). These findings of epithelial damage were confirmed by the presence of disrupted epithelium in H&E stained histology (Figure 1). It is interesting that CFM detected changes with the subclinical concentration of BZK (0.02%), particularly given that in a recent study changes in the ovine epithelium due to 0.02% BZK were not detected by colposcopy [7]. Both CFM and histology from animals treated with HEC were comparable to those of PBS treated animals. HEC gel is recognized as a nontoxic topical agent and is widely used as a universal placebo in microbicide safety studies [16]. Repeated dosing with HEC for seven days resulted in no discernable difference from untreated controls by histology in mice [18].

A critical aspect of this study was the development of an algorithm based on a mean-to-median object size analysis to measure epithelial damage in confocal micrographs. Observation of confocal micrographs in which epithelial damage occurred as confirmed by histology revealed the increased presence of aggregated material or nuclei that could not be clearly differentiated from neighboring nuclei. Preliminary tests (not shown) indicated that although visually there was an increase in staining pattern with damage, the degree of damage did not necessarily relate with percent area of staining. Thus we chose a metric that would take into account the fact that images of epithelium went from uniformly sized objects (single nuclei) to larger objects when degree of staining increased and individual nuclei could not be readily separated. The quantitative algorithm based on the mean-to-median object size assessment provided discriminatory information regarding level of damage induced by the applied agents and results were consistent with histology findings, validating the ability of this technique to detect microbicide-induced changes in epithelial morphology. We chose to image *ex vivo *specimens to facilitate development of an automated algorithm for determination of damage to the epithelium. We intend to expand these studies for real time *in vivo *and longitudinal evaluation of microbicide toxicity in the ovine model. It is expected that many more sites can be assessed by CFM than is feasible to biopsy in chronic studies.

Another advantage of CFM is that it can be coupled with any number of fluorescent indicators of cellular or molecular injury. It is noted that the ensuing analysis of confocal micrographs and assessment of damage is dependent upon the fluorophore used for image contrast and localization and staining patterns. PI stains nuclei of damaged cells. Other fluorescent dyes typically used with CFM are nonspecific in that they either stain all nuclei (e.g. acriflavine hydrochloride) or both intracellular and extracellular features (e.g. fluorescein) [8]. Assessment of damage in these cases may be based on morphology changes such as in a study by Bott et al., evaluating differences in morphology of the ovine epithelium due to hormonal treatment that used acriflavine orange for contrast [15]. An interesting possibility in preclinical studies may be the use of molecular-specific fluorophores as is being pursued in gastrointestinal imaging [19,20], that indicate inflammation or damage that does not result in cell lysis. Several biomarkers of inflammation related to microbicide safety assessment are being investigated in vaginal mucosa [21,22] and could in the future be targeted with fluorophores for imaging with CFM. The ability of CFM to detect cellular/molecular indicators of damage will in part depend on location of fluorescent signals. The system utilized in this study is restricted to signals at or near the surface (working distance from 0-80 μm depending on the imaging probe used) with a similar clinical version allowing maximal depth at 130 μm [8]; CFM with dynamic depth-sectioning is also available providing optical sections to depths 250 μm using larger diameter imaging probes (12.8 mm), that are suitable for large animal and human studies, with the advantage of improved resolution [8] but the challenge of determining the actual depth being imaged when using a handheld probe.

CFM could potentially be adapted to clinical studies since the imaging fiber is small, can be easily placed in the vaginal tract, and the system is commercially available, however a requirement for clinical translation is that suitable non-toxic fluorophores be available. Fluorophores used in human GI tract imaging include fluorescein and acriflavine hydrocholoride [8-14]. No live/dead cell (fluorescent) dyes are known to be approved for clinical use. In the cervicovaginal or rectal tract, fluorescein could be readily applied and has wide acceptance for clinical use in various tissues (it is FDA approved for angiography of the retina) [11,12]. Currently, the possibility of quantitative CFM imaging using fluorescein for contrast is being investigated by our group in the ovine model.

## Conclusion

The results of this study indicate that CFM could be a useful tool for detecting microscopic surface effects of candidate microbicides on the vaginal epithelium. Agents in solution and gel formulations were tested successfully; gel formulations did not interfere with imaging. Results were consistent with those of histology and with findings reported in the literature for each of the agents studied. Future efforts will include translation of the techniques into *in vivo *studies and evaluation of the methods for use with microbicide candidates that are believed to have minimal cytotoxic effects but may nonetheless alter the vaginal epithelium.

## Competing interests

The authors declare that they have no competing interests.

## Authors' contributions

GV, study concept and design, acquisition of data, algorithm conceptualization with IP, analysis and interpretation of data, statistics, drafting of the manuscript. KV, study concept and design, coordination and acquisition of samples, interpretation of data, drafting of manuscript. MM, study concept and design, interpretation of data, provided expertise on imaging/optics, drafting of the manuscript. NB, study concept and design, analysis and interpretation of data, expertise on microbicides and epithelial injury, drafting of the manuscript. IP, image processing and algorithm development, data analysis. JW and BBell, sample collection, acquisition of data, data analysis and scoring. All authors read and approved the final manuscript.

## Pre-publication history

The pre-publication history for this paper can be accessed here:

http://www.biomedcentral.com/1471-2334/12/48/prepub
